# Unravelling the mystery of maxillary sinus malignancy initiated with periapical radiograph: A case report

**DOI:** 10.6026/9732063002001780

**Published:** 2024-12-31

**Authors:** Azeeja Parayil Ajma, Sunila Thomas, Snifa Velayudhapanicker Sundaresan, Jeena Raj

**Affiliations:** 1 Department of Oral Medicine and Radiology, PMS College of Dental Science and Research, Kerala University of Health Sciences, Trivandrum, India

**Keywords:** Carcinoma, maxillary sinus malignancy, periapical radiograph, intraoral periapical radiograph, tumour

## Abstract

Maxillary sinus malignancies, rare but often mimic dental infections, can present as asymptomatic growth until perforation. Symptoms mimic inflammatory sinus lesions, with many patients seeking dental treatment first. Clinical presentation depends on which walls of sinus is the affected. Hence, we report the diagnostic journey of a patient, with odontogenic symptoms, ultimately diagnosed as malignancy of the maxillary sinus. The diagnosis initiated from an intraoral periapical radiograph (IOPAR) in this case emphasizes the significance of imaging in early detection of maxillary sinus malignancies, masquerading as dental infection.

## Background:

Maxillary sinus malignancies are asymptomatic initially, diagnosed only in the advanced stage with poor prognosis [1]. 3% of head and neck malignancies involve the maxillary sinus with an annual incidence of 0.5-1.0 case per 100,000 populations [2]. Risk factors include tobacco smoke and chronic inflammation of maxillary sinus [3]. These tumors present nonspecific symptoms, but characteristic imaging features aid in the diagnosis [4]. The radiological differentiation of sinus malignancies pose a challenge to the clinician due to similarity in imaging features, tumor location, invasion into adjacent bone and anatomical structures. Hence, we report a case which may have gone unidentified but diagnosed based on remarkable finding initiated from an intraoral periapical radiograph correlated with clinical symptoms.

## Case presentation:

**Figure 1:**
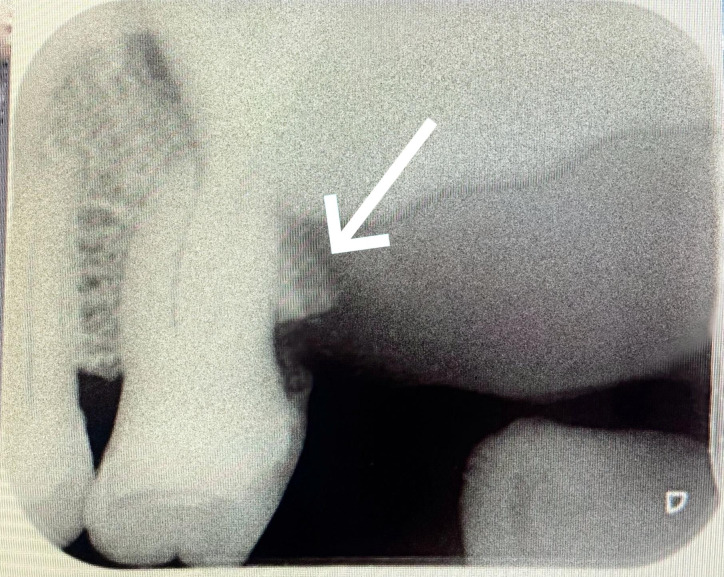
IOPAR showing alveolar bone destruction distal to 26.

**Figure 2:**
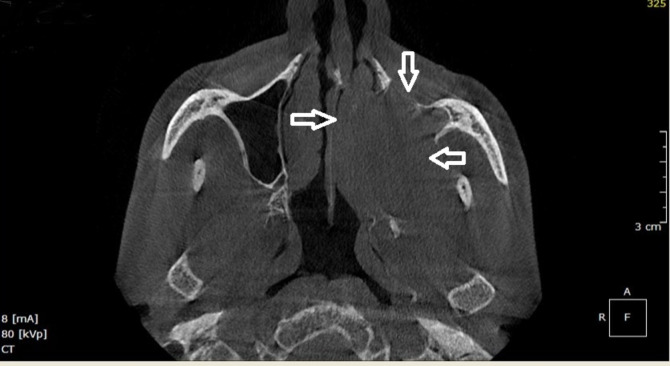
CBCT - Axial view shows radiopacity on left maxillary sinus, destruction of anterior, anterolateral and medial walls with extension into left nasal cavity.

**Figure 3:**
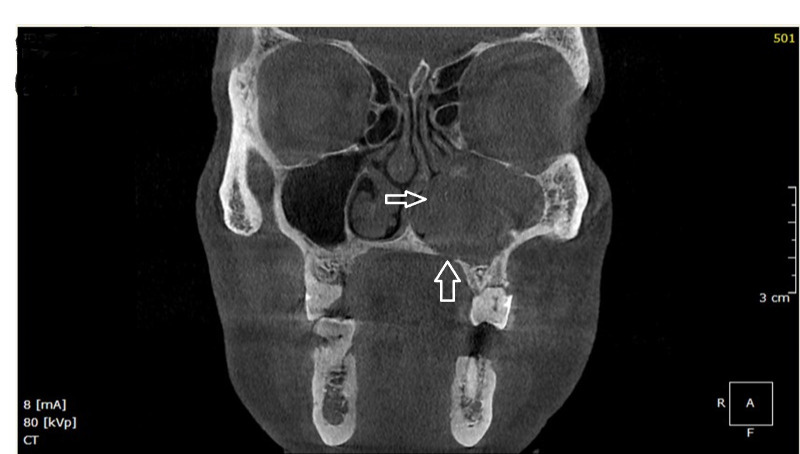
CBCT - Coronal view shows erosion of nasal floor, anterolateral wall and medial wall of maxillary sinus with extension into nasal cavity.

**Figure 4:**
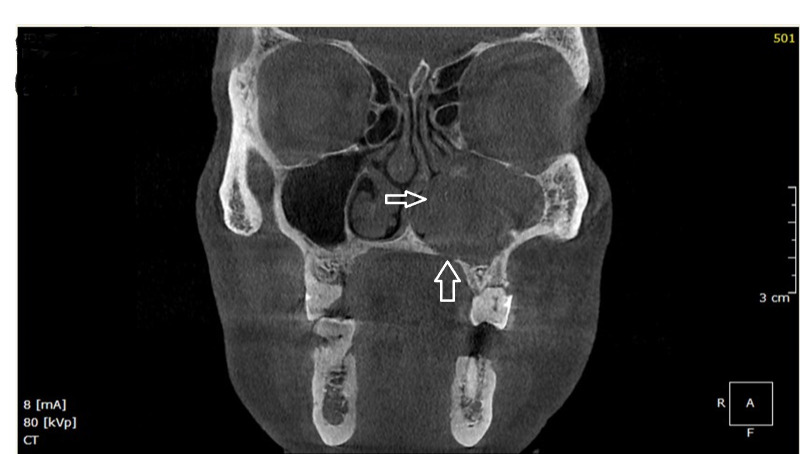
Sagittal View - Erosion of alveolar bone distal to 26 with disruption of floor of maxillary sinus and root resorption 26 Intraoral and CBCT imaging were suggestive of malignancy involving the left maxillary sinus.

**Figure 5:**
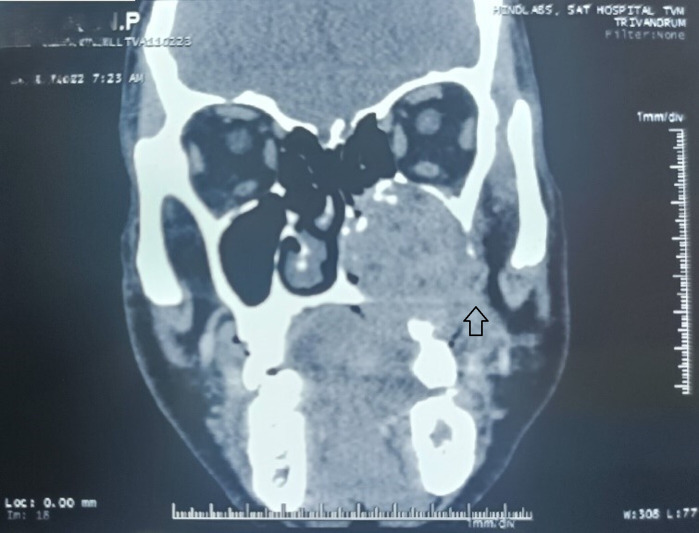
CECT - Coronal views shows focal erosion in anterior wall of left maxillary sinus noted with no intra orbital or intra cranial extension.

63-year-old male patient reported to the Department of Oral Medicine and Radiology with palatal swelling and pain on the extraction site of upper left posterior teeth done one year back. The teeth 27, 28 were extracted due to mobility and were uneventful. The patient gave a history of pain and swelling on the site after extraction and numbness on the left side of face and left nasal region for one year. He was an outdoor worker, a truck driver by profession. He was a Diabetic under medication and his glycaemic status was well controlled. He was a chronic smoker with alcoholism till 2 years ago. There was no evident extra-oral swelling, no epistaxis, epiphora or nasal voice. There was also paraesthesia on left malar region and lateral aspect of nose. Left cervical II A lymph nodes were palpable. On intra oral examination a diffuse erythematous swelling of size 2 x 3 cm noted on the posterior palatal mucosa in relation to edentulous 27, 28 regions. Intra oral periapical radiograph revealed destruction of alveolar bone distal to 26 regions. The irregular destruction of bone in this region made the diagnostic turning point to this case (Figure 1). Maxillary lateral occlusal topography also revealed extensive alveolar bone destruction apical and distal to 26 involving the posterior hard palate. A panoramic radiograph was taken to reconfirm and assess any further sites of bone loss in the oral cavity and it revealed extensive horizontal bone resorption and irregular bone destruction in the edentulous alveolar ridge corresponding to 27, 28 regions, with disruption of floor of maxillary sinus. CBCT view revealed opacification of left maxillary sinus by extensive radio-density comparable to soft tissue density. Axial view shows radiopacity on left maxillary sinus, destruction of anterior, anterolateral and medial wall with extension into left nasal cavity (Figure 2).Coronal view shows extensive radiopacity filling the left maxillary sinus with destruction of nasal floor, anterolateral wall and medial wall of maxillary sinus, with intact orbital floor (Figure 3). Erosion of alveolar bone distal to 26 with disruption of floor of maxillary sinus and root resorption 26 in Sagittal view (Figure 4).To evaluate the extent of lesion into adjacent structures, contrast enhanced Computed Tomography (CECT) was done which revealed a well-defined lobulated heterogeneous soft tissue mass in left maxilla, measuring 5.1 x 4.1 x 3.5 cm. Lesion caused destruction of left upper alveolus at the level of molar teeth, left side of hard palate, medial and postero-lateral walls of left maxillary sinus. Lesion filled the left maxillary sinus with extension into left nasal cavity, pterygopalatine fossa and masticator space with erosion of medial pterygoid process with invasion to medial pterygoid muscle and inferior aspect of temporalis. Focal erosion in anterior wall of left maxillary sinus was noted with no intra orbital or intra cranial extension (Figure 5). The CT was suggestive of malignant neoplasm centred in left maxillary sinus. Location of lesion in the posterior palate also considered the possibility of salivary gland malignancy extending into the maxillary sinus. Biopsy and microscopic evaluation was done. A final diagnosis of carcinoma of the left maxillary sinus was given based on clinical and radiographic features coupled with microscopy.

## Discussion:

Carcinoma of the Maxillary sinus, a neoplasm with an insidious onset, is most often diagnosed only in the advanced stage of disease. Maxillary sinus lesions present non-specific symptoms initially, mimicking odontogenic infections, nasal, lacrimal or sinus inflammations [5]. About 40% to 60% of cases present with facial asymmetry, intraoral swelling and tumor extension into the nasal cavity [6]. Maxillary sinus carcinomas are twice common in men with 95% cases above 40 years [7].The present case reported with persistent pain and palatal swelling following teeth extraction. Maxillary sinus malignancies demonstrate characteristic clinical manifestations. Absence of epistaxis, epiphora, diplopia and paraesthesia excludes the possibility of a malignant tumor [8, 9]. This case was diagnosed only in the late stage of disease, with paresthesia as a classic sign indicating malignancy. Large air space in the maxillary sinus facilitates undisturbed growth of tumor, with symptoms manifested following erosion of the walls [2]. Destruction of medial wall of sinus causes nasal obstruction, discharge and epistaxis. Dental signs are manifested when floor of the sinus is eroded and presents as pain, swelling of palate or alveolar ridge and mobile tooth. Involvement of lateral wall of sinus causes facial and vestibular swelling, while superior extension causes protrusion of eyeball and diplopia [2]. Lymph node metastases are uncommon in sinus malignancy. The left cervical II A lymph nodes palpable in the present case may are due to invasion of the tumor into the oral cavity, which is rich in lymphatic network. Majority of patients with carcinoma of maxillary sinus are diagnosed in the late stage. Our patient had paresthesia in the malar region, which was earlier misinterpreted as post extraction complication, hence the delay in diagnosis. Paresthesia is a reliable indicator of malignancy, though it occurs in post-surgical nerve injury. Hence, it is mandatory that the possibility of a malignant neoplasm be ruled out in all patients presenting with paresthesia [8].

The conventional radiograph, IOPAR depicted the destructive lesion involving the alveolar bone which led to further investigations. Destruction of walls of maxillary sinus, a clear sign of malignancy was evident in CBCT. Tumor extension into adjacent structures was confirmed in CT which also ruled out intracranial extension in this case. More than 70% to 90% of cases of maxillary sinus carcinomas detected with CT shows bony destruction [10]. Differential diagnosis of maxillary sinus carcinoma includes primary sinonasal neoplasms like undifferentiated carcinoma, nasopharyngeal carcinoma, lymphoma and adenocarcinoma of minor salivary gland origin, metastatic diseases [11]. Carcinomas of maxillary antrum seem to present a more aggressive behaviour than those of the salivary gland tumors. The risk factors associated with maxillary sinus carcinoma include chronic exposure to chemicals such as nickel, chlorophenol, formaldehyde, textile dust, wood and cigarette smoking [7, 12 and 13]. Our patient, a chronic smoker, was an outdoor worker with possible exposure to environmental pollutants. Surgery followed by radiotherapy remains the treatment of choice for Maxillary sinus malignancies [14]. The treatment outcome and prognosis depend on the tumor stage, histopathology and invasion into vital structures [15]. Our patient presented with symptoms mimicking dental infection and undergone extraction of mobile upper left molars with persistent symptoms even one year after extraction. This case was initially mistaken as residual infection, though not supported by a radiographic evaluation earlier. Large air-filled maxillary sinus allows room for asymptomatic expansion of tumor often diagnosed late when walls of the sinus are eroded. In this case there was erosion of medial wall with extension into the nasal cavity; paraesthesia over left nasal cavity was a reliable indicator of malignancy, though there was no epistaxis or nasal voice. Numbness over the left malar region explains perforation of anterior wall. Destruction of maxillary sinus floor in this patient simulated dental symptoms, which was diagnosed promptly with IOPAR depicting the irregular destruction of alveolar bone.

## Conclusion:

Maxillary sinus carcinomas with non-specific clinical symptoms have poor prognosis due to delay in diagnosis. Most often masquerading as dental infections, sinus malignancies may be misdiagnosed by the dental professional unless a thorough clinical and radiographic evaluation is done. Hence, the importance of standard imaging techniques such as periapical radiography in early recognition of clinically quiet malignant lesions of maxillary sinus is highlighted.

## Financial support and sponsorship:

Nil

## Competing Interests:

None
